# The miR-142 Suppresses U-87 Glioblastoma Cell Growth by Targeting EGFR Oncogenic Signaling Pathway

**DOI:** 10.22037/ijpr.2021.115089.15193

**Published:** 2021

**Authors:** Fatemeh Gheidari, Ehsan Arefian, Fatemeh Jamshidi Adegani, Fereshteh Fallah Atanaki, Masoud Soleimani

**Affiliations:** a *Department of Biotechnology, College of Science, University of Tehran, Tehran, Iran. *; b *Stem Cell Technology Research Center, Tehran, Iran. *; c *Department of Microbiology, School of Biology, College of Science, University of Tehran, Tehran, Iran. *; d *Pediatric Cell Therapy Research Center, Tehran University of Medical Sciences, Tehran, Iran. *; e *Laboratory for Stem Cell and Regenerative Medicine, Natural and Medicinal Sciences Research Center, University of Nizwa, Nizwa, P. O. Box: 33, PC 616, Oman. *; f *Laboratory of Complex Biological Systems and Bioinformatics (CBB), Department of Bioinformatics, Institute of Biochemistry and Biophysics (IBB), University of Tehran, Tehran, Iran. *; g *Department of Hematology, Faculty of Medical Sciences, Tarbiat Modares University, Tehran, Iran.*

**Keywords:** miR-142, glioblastoma, U87, apoptosis, AKT1

## Abstract

Glioblastoma is the most lethal malignancy of the brain and is resistant to conventional cancer treatments. Gene-therapy approaches like using tumor suppressor miRNAs are promising in the treatment of glioblastoma. They control the expression of oncogenes and influence tumor features and behaviors. Therefore, in the present study, it was predicted that miR-142 regulates oncogenic epidermal growth factor receptor (*EGFR*) signaling pathway via TargetScan and miRWalk online tools. Its differential expression level was reduced in glioblastoma according to the previous microarray results, and its predicted target genes were upregulated, as shown by the Expression Atlas. The miR-142 was overexpressed in U-87 glioblastoma cells via lentiviral transduction, and the way it influences proliferation and migration of cells was investigated through MTT assay and wound healing assay. Apoptosis rate was also measured via the Annexin V assay, and cell-cycle analysis was done. Then, real-time polymerase chain reaction (real-time PCR) and western blotting were performed to assess fold changes in mRNA and protein levels of the miR-142 predicted targets. Direct target genes of miR-142 were confirmed through a dual-luciferase reporter assay. The miR-142 significantly suppressed cell proliferation and migration and induced apoptosis and cell-cycle arrest in U-87 glioblastoma cells. This was accompanied by a decrease in expression of SHC adaptor protein 4 (*SHC4*), phosphatidylinositol-4,5-bisphosphate 3-kinase catalytic subunit alpha (*PIK3CA*), v-akt murine thymoma viral oncogene homolog 1 (*AKT1*), Kirsten rat sarcoma viral oncogene homolog (*KRAS*), and mitogen-activated protein kinase 8 (*MAPK8*) oncogenes at mRNA and protein levels in glioblastoma cells. Also, *AKT1* was demonstrated as a direct target of miR-142. Overall, miR-424 acts as tumor suppressor miRNA in glioblastoma cells.

## Introduction

Glioblastoma multiform (GBM) is the deadliest malignancy in the central nervous system (1, 2). Due to reasons, such as being surrounded by the blood-brain barrier and being so aggressive caused by the mobile nature of glial cells, it has a poor prognosis for conventional treatments of cancer, surgery, chemotherapy, and radiotherapy, with a median survival rate of 9-19 months (3, 4). Scientists have made great efforts to use novel therapeutic strategies to cure glioblastoma by trying to make glioblastoma-provoking molecular mechanisms better known (5, 6). The epidermal growth factor receptor (*EGFR*) signaling pathway is overactive in about 80% of patients with GBM and induces cell growth, proliferation, and migration (7, 8). This may be as a result of mutations in its receptor tyrosine kinases (*EGFR*-45%, Erb-B2 receptor tyrosine kinase 2 (*ERBB2*)-8%, platelet-derived growth factor receptor alpha (*PDGFRA*)-13%, and *MET*-4%), core oncogenes (*RAS*-2%, *PI3K*-15%, and *AKT*-2%), or tumor suppressors (phosphatase and TENsin homolog (*PTEN*)-36%, neurofibromatosis type 1 (*NF1*)-18%, and forkhead box O (*FOXO*)-1%) in patients with GBM (7).

The miRNAs are small non-coding RNAs, regulating gene expression at the mRNA level via binding to complementary regions in 3’ untranslated regions of their target genes (9). They play a crucial role in adjusting many physiological conditions like growth and differentiation and their dysregulation leads to pathological circumstances (10, 11). Various candidates of miRNAs are in the potential list of tumor suppressor miRNAs and have been investigated in clinical trials for curing glioblastoma or other types of cancer (12-14).

In this research, the effect of miR-142 on U-87 glioblastoma cells was studied. For the first time, miR-142 was introduced as a suppressor of several oncogenes of the *EGFR* signaling pathway *in-silico,* and then, its role was also checked *in-vitro*. U-87 cells carry mutations on *NF1* and *PTEN* genes that are inhibitors of Kirsten rat sarcoma viral oncogene homolog (*KRAS*) and phosphatidylinositol-4,5-bisphosphate 3-kinase catalytic subunit alpha (*PIK3CA*) oncogenes at downstream of *EGFR* (15). Following these mutations, *KRAS* and *PIK3CA* activate their downstream signaling pathways out of control and cause immoderate cell division and survival in glioblastoma cells (7, 8). Meanwhile, considering the regulation of these oncogenes through miR-142, inhibition of proliferation and migration of U-87 cells was expected *in-vitro*.

## Experimental

*Prediction of the miRNA and target genes*

TargetScan (www.targetscan.org) and miRWalk 2.0 (zmf.umm.uni-heidelberg.de › mirwalk2) online tools were used to find tumor suppressor miRNAs suppressing several oncogenes of the EGFR signaling pathway (Table 1). After choosing miR-142, its expression was investigated in tumor tissues of patients with GBM versus normal tissues through a microarray dataset (GSE90603) from the Gene Expression Omnibus (www.ncbi.nlm.nih.gov › geo) database. At the next step, expression of miR-142 predicted target genes was assessed in patients with GBM on the Expression Atlas (www.ebi.ac.uk › gxa). Their mutations or expression changes that had occurred in glioblastoma were also looked up via the Cancer Genome Atlas (www.cancer.gov › research › structural-genomics › tcga).

*Cloning of miRNA gene*

 The miR-142 locus was found in the miRBase (www.mirbase.org) database. The locus was amplified by the polymerase chain reaction (PCR) technique on the genomic DNA template through designing specific primers. The miR-142 amplicon was inserted into the cloning site of the pCDH-GFP-Puro lentiviral vector (System Biosciences) by cutting with *EcoRI* and *BamHI* restriction enzymes (Thermo Fisher Scientific).

*Cell culture*

U-87 MG and HEK293T cell lines were provided and fully characterized by the Iranian Biological Resource Center. Both cell lines were cultured in Dulbecco’s Modified Eagle’s medium (Gibco) supplemented with 10% Fetal Bovine Serum (Gibco) and were incubated at 37 °C with 5% CO_2_ and 95% of humidity.

*Virus packaging and transduction*

For viral packaging, the pCDH vectors containing miR-142 and scrambled were co-transfected to HEK293T cells by psPAX2 and pMD2.G helper vectors (System Biosciences), using poly ethylene imine transfection reagent (Sigma). Cell culture media was changed every 24 h with fresh media, and virus-containing supernatant was collected and stored in the fridge for 4 days. Then, the supernatant was filtered with 0.2 µm sterile filters and was kept at -80 °C freezer. U-87 cells were seeded in an appropriate culture plate with 40% of confluency one day before transduction. Virus-containing supernatants were mixed with fresh media 1:1 and added to cells. To improve transduction efficiency, 10 µg/mL of polybrene (Sigma) was added to cells. The rate of transduction was checked via fluorescent microscopy 48 h after transduction.

*MTT proliferation test*

Transduced U-87 cells were seeded in a 96-well sterile culture plate at a density of 8 10^3^ cells/well and were cultured for 72 h at a 37 °C CO2 incubator. Furthermore, the culture media was removed, and 100 µL of fresh media containing 0.5 mg/mL of 3-(4,5-dimethylthiazol-2-yl)-2,5-diphenyltetrazolium bromide (Sigma) was added to each well. The plate was incubated for another 4 h in the dark. Then, the media was removed, and the crystals formed at the bottom of the wells were dissolved in dimethyl sulfoxide by shaking and pipetting. Finally, absorbance at 590 nm was measured by a plate reader (BioTek).

*Scratch wound assay*

Transduced U-87 cells were seeded in a 12-well plate at a density of 10^5^ cells/well with 80% of confluency. The next day, a vertical and a horizontal line was drawn on the bottom of each well by a sterile 1-10 µL pipette tip, then the media was removed, and the cell surface was washed with phosphate buffer saline (PBS). Then, the complete media was added to cells and an image was taken by a digital camera at 0, 24, 48, and 72 h from the same view of the scratch while culturing. Image J software was employed to measure the distance of the cells.

*Annexin V-PE/ 7AAD apoptosis test*

Transduced U-87 cells were seeded in a 24-well sterile culture plate at a density of 410^4^ cells/well and were cultured for 72 h at a 37 °C CO2 incubator. Then, cells were dissociated with trypsin in 1.5 mL microtubes and were rinsed twice by a binding buffer of the Annexin V-PE/ 7AAD apoptosis kit (BD Biosciences). The cell pellet was dissolved in 200 µL of binding buffer, and then, 5 µL of Annexin V-PE was added to each microtube following incubation for 15 min in the dark for staining. Next, the cells were washed, 5 μL of 7AAD dye was added, and they were assessed by flow cytometry (BD Biosciences). FlowJo 7.6.1 software was employed to analyze flow cytometry data.

*Cell-cycle test*

Transduced U-87 cells were seeded in a 24-well sterile culture plate at a density of 410^4^ cells/well and were cultured for 72 h at a 37 °C CO2 incubator. Then, the cells were dissociated with trypsin in 1.5 mL microtubes and were rinsed once with PBS. Harvested cells were added to 70% ethanol-containing microtubes dropwise on vortex and were kept overnight in -20 °C freezer. In the next day, ethanol was removed by centrifuge, and the cell pellet was dissolved in 200 μL of a solution containing 50 μg/mL of Propidium Iodide (Sigma), 1 mg/mL of RNase (Thermo Fisher Scientific), and Tryton X-100 (Sigma). Cells were incubated for 40 min in the dark at 37 °C, and then they were measured by flow cytometry. FlowJo 7.6.1 software was employed to analyze flow cytometry data. 

*Gene expression analysis using real-time PCR*

The total RNA was extracted using TRIzol (Invitrogen) reagent from the transduced U-87 cells at 72 h, according to the manufacturer’s instructions. For investigation of miRNA expression, stem-loop RT primers, as well as forward and universal reverse primers, were designed using the method described previously (16) for *miR-142* and *SNORD47* as an internal control (Table 2). For investigating the expression of target genes, random hexamer was used for cDNA synthesis, and specific primers were applied for *KRAS*, *v-akt murine thymoma viral oncogene homolog 1 (AKT1), PIK3CA, SHC adaptor protein 4 SHC4 (SHC4), mitogen-activated protein kinase 8 (MAPK8), and β2 microglobulin (β2M) *as an internal control (Table 3). Real-time PCR was done with an ABI StepOnePlus system and SYBR Green Master mix (Ampliqon) under the following condition: 2 min at 95 °C and 40 cycles by 10 s at 95 °C, and 30 s at 62 °C following the melt curve program. The 2^-ΔΔCt^ method was employed to analyze the data.

*Western blotting*

The transduced U-87 cells were lysed on ice using radioimmunoprecipitation assay (RIPA) buffer and protease inhibitor cocktail (Merck) at 72 h. After 30 min, the cells’ protein extract was isolated by centrifuge, was loaded into a 10% sodium dodecyl sulfate-polyacrylamide gel electrophoresis SDS (SDS-PAGE) system, and was separated via electrophoresis at the voltage of 150 V. Then, the samples were transferred to a polyvinylidene fluoride (PVDF) membrane, followed by membrane blocking with 5% skim milk (Merck). Primary antibody was incubated with anti‐KRAS (1:200, Sigma), anti‐AKT1 (1:2500, Abcam), anti‐PIK3CA (1:5000, Abcam), anti‐SHC4 (1:500, Sigma), anti‐MAPK8 (1:1000, Sigma), and anti‐β-Actin (1:1000; Abcam). PBST buffer was used to rinse the membrane. Then, the membrane was incubated in the horseradish peroxidase-conjugated secondary antibody (1:1000; Abcam) solution for 4 h. The enhanced chemiluminescence (ECL) Western Blot Substrate (Thermo Fisher Scientific) was added in a dark room followed by photography. The GelAnalyzer software 2010a was employed to investigate the relative density of bands, and they were normalized with β-Actin.

*Dual-luciferase reporter test*

The 3’ untranslated region of the *AKT1* gene was inserted into the Dual-luciferase reporter psiCHECK 2.0 vector (Promega) under the Renilla luciferase coding region with *T4 Ligase* enzyme (Thermo Fisher Scientific). The HEK293T cells were seeded in a 96-well plate at a density of 10^4^ cells/well. In the next day, cells were cotransfected with pCDH-miR-142/pCDH-scrambled and psiCHECK-AKT1-3’UTR/ psiCHECK-control vectors using polyethylenimine (PEI) transfection reagent (Sigma). At 48 h, luciferase activity was measured using the Dual‐Luciferase Reporter Assay System kit (Promega) according to the manufacturer’s protocol. Firefly luciferase activity was employed to normalize the Renilla luciferase activity.

*Statistical analysis*

All the experiments were performed in triplicate. After analyzing measurements, GraphPad Prism 8 software was employed to calculate mean, and standard deviation among biological repeats, and the Student’s *t*-test was used to define the significance of changes between treatment and control groups, and graphical charts were drawn.

## Results

*Results of in-silico study suggested downregulation of miR-142 in glioblastoma and upregulation of its predicted targets from the EGFR signaling pathway*

**Figure 1:**
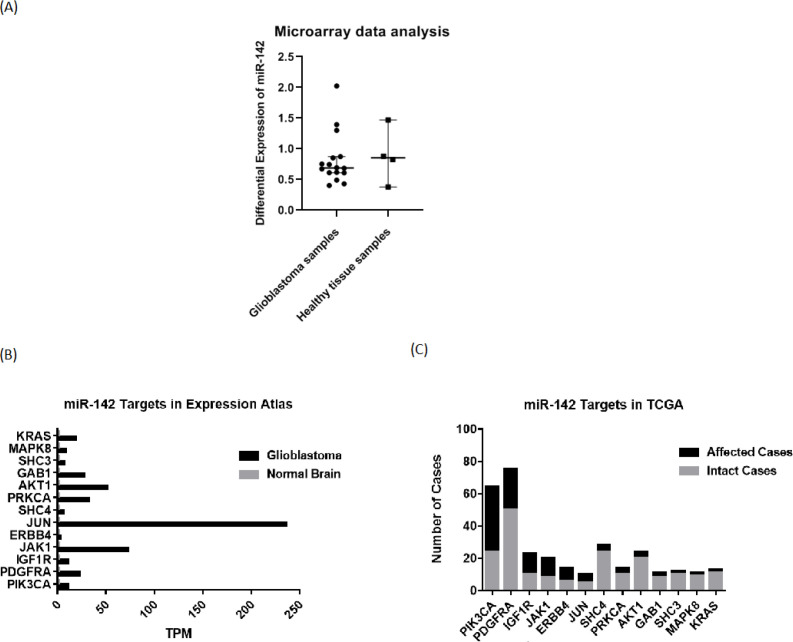
*Bioinformatic investigation of *miR-142* level and changes in its predicted target genes in glioblastoma. (A) The GSE90603 microarray dataset was provided that compares miRNA profiles of glioblastoma samples and healthy tissue samples in patients with GBM from the GEO database. Medians and 95% confidence intervals are *specified *in the graph. (B) According to the Expression Atlas website, the next-generation sequencing (NGS) data on expression levels of miR-142 predicted target genes were extracted. (C) The data from the TCGA website show that all the predicted target genes of miR-142 are altered in some cases of glioblastoma. GEO: Gene Expression Omnibus; TCGA: The Cancer Genome Atlas; NGS: next-generation sequencing*

For investigating expression changes of miR-142 in patients with GBM, they were checked through available microarray and sequencing data on the GEO database. Analyzing the data from a relevant study (17) (GSE90603 dataset) on 16 glioblastoma tumor samples and 4 standard samples from patients with GBM by GEO2R, it was found that the median differential expression level of miR-142 was equal to 0.687933 in glioblastoma tumor samples compared to 0.852646 in healthy tissue samples (Figure 1A). So, the median differential expression level of miR-142 was found to be lower in patients with GBM. For studying changes in miR-142 predicted targets in glioblastoma, first, their expression level was checked in sequencing data on the Expression Atlas database. Then, their mutations or expression changes were assessed on the TCGA database. It was found that most miR-142 predicted targets are overexpressed in the glioblastoma state compared to the normal brain state. For most of the genes, the expression level was “High” or “Medium” in the glioblastoma state *vs.* “Under-cutoff” in normal brain state. Also, it was observed that most of the miR-142 predicted target genes are mutated along with a change in their copy number in glioblastoma, suggesting their role in the progression of glioblastoma tumor (Figures 1B-1C).

*The miR-142 inhibited proliferation and migration of U-87 glioblastoma cells*

**Figure 2:**
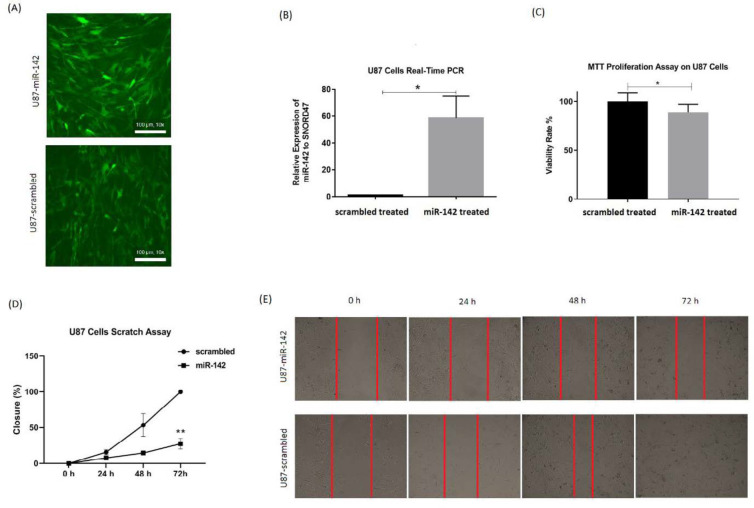
*The miR-142 Overexpression in U-87 cells and the way it influences cells’ proliferation and migration rate. (A) Fluorescent microscopy shows the expression of GFP reporter protein in almost all the cultured U-87 cells after lentiviral transduction of miR-142 and scrambled. (B) Significant overexpression of miR-142 in the transduced U-87 cells was confirmed via real-time PCR. (C) The miR-142 transduced U-87 cells show significantly less proliferation rate than scrambled transduced cells at 72 h. (D and E). Migration rate was significantly lower in miR-142 transduced cells in comparison with the scrambled transduced group at 72 h in the scratch wound assay. Data are shown as mean ± SD of biological repeats (* ^*^P < 0.05, ^**^P *< 0.01). GFP: green fluorescent protein; MTT: 3‐(4, 5‐dimethylthiazol‐2‐yl)‐2, 5‐diphenyltetrazolium bromide; SD: standard deviation*

For evaluating miR-142 effects on glioblastoma tumor cells, MTT test and wound healing test were done for U-87 cells at 72 h after transduction of lentiviruses containing miR-142/ scrambled. The rate of transduction was checked through observation of green fluorescent protein (GFP) via fluorescent microscopy (Figure 2A). Also, real-time PCR was used to show miR-142 overexpression relative to *SNORD47* internal control in the transduced U-87 cells. The miR-142 was 58.5 ± 16.3 (^*^P < 0.05) folds more expressed in miR-142 transduced cells compared to the scrambled transduced control (Figure 2B).

The results of the MTT assay showed that the viability rate in U-87 cells transduced with miR-142 was significantly decreased (^*^P < 0.05) compared to the scrambled control. Viability ratio in U-87 cells transduced with miR-142 was 88.7 ± 16.4% of the control group (Figure 2C). Besides, the scratch assay results approved that miR-142 significantly inhibited migration (^**^P < 0.01) in glioblastoma cells at 72 h after transduction. The closure percentage of miR-142 transduced cells, was 7.8 ± 1.2% at 24 h, 14.7 ± 1.2% at 48 h, and 27.6 ± 7.3% at 72 h *vs*. 15.8 ± 3.5%, 53.6 ± 16.1%, and 100 ± 0% for the control group at 24, 48, and 72 h, respectively (Figures 2D-2E). So, it was shown that miR-142 significantly suppressed proliferation and migration in glioblastoma cells.

*The miR-142 induced apoptosis and cell-cycle arrest in U-87 glioblastoma cells*

**Figure 3:**
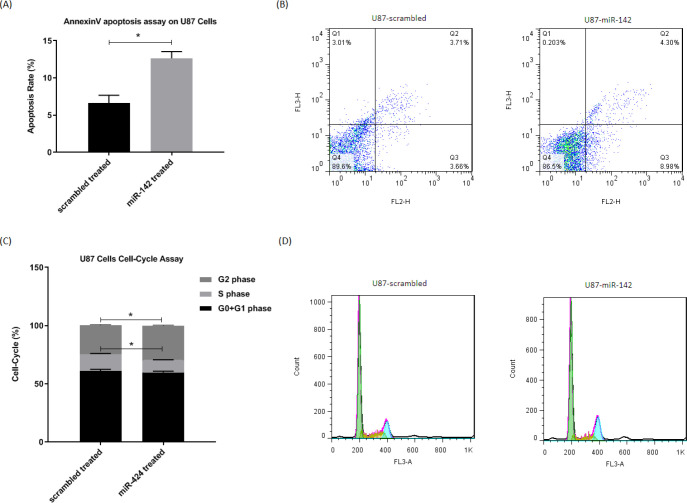
*The miR-142 overexpression causes apoptosis and cell-cycle arrest in U-87 cells. A, B. Flow cytometry results show a significantly higher number of apoptotic cells at 72 h after transduction by miR-142 in comparison with the control group in Annexin V-PE/7AAD assay. C, D. Cell-cycle analysis at 72 h after transduction revealed that miR-142 transduced U-87 cells are significantly less at the S phase, and they are arrested at the G2 phase compared to the control group. Data are shown as mean ± SD of biological repeats (* ^*^P* < 0.05). SD: standard deviation*

The way miR-142 influences apoptosis and cell cycle in glioblastoma U-87 cells was evaluated via flow cytometry. For evaluation of apoptosis, the Annexin V-PE/7AAD apoptosis test kit was used at 72 h after transduction, and the results showed that miR-142 significantly induced apoptosis (^*^P < 0.05) in U-87 glioblastoma cells (Figure 3A-3B). The cell-cycle analysis of U-87 cells was also done via fixing them and PI staining at 72 h after transduction. The flow cytometry results showed a significantly higher number of cells (^*^P < 0.05) at the G2 phase with fewer cells at the S phase in miR-142 transduced cells than the scrambled control. In miR-142 transduced cells, 59.9 ± 1.0%, 10.6 ± 0.3%, and 29.4 ± 0.6% of cells were at G0+G1, S, and G2 phases, while in the scrambled transduced cells, 61.1 ± 1.4% of cells were at G0+G1, 14.5 ± 0.6% of cells were at S phase, and 24.9 ± 0.5% of cells were at G2 phase (Figures 3C-3D). Altogether, miR-142 significantly induced apoptosis and cell-cycle arrest in U-87 glioblastoma cells.

*The miR-142 regulated expression of several oncogenes of the EGFR signaling pathway*

**Figure 4:**
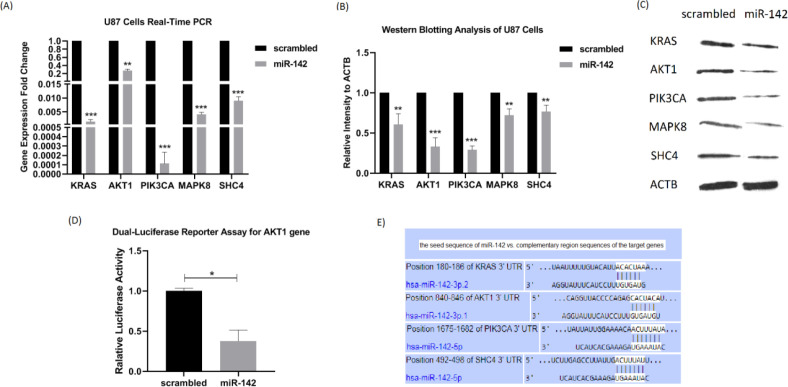
The miR-142 overexpression downregulates the expression of its predicted target genes. (A) At 72 h after transduction, changes in the mRNA expression level of 5 predicted target genes of miR-142 were investigated in U-87 cells by real-time PCR. *KRAS*, *AKT1*, *PIK3CA*, *MAPK8*, and *SHC4* were all significantly downregulated. (B and C) The results of the western blotting analysis showed a significant decrease in* the *protein expression level of KRAS, AKT1, PIK3CA, MAPK8, and SHC4 at 72 h after transduction by miR-142 in comparison with the control. (D) The miR-142 directly targets AKT1 mRNA. Dual-luciferase reporter assay shows a significant decrease (0.38 ± 0.13) in light intensity resulting from luciferase enzyme activity cloned at upstream of AKT1 3’-UTR in psiCHECK2.0 vector. (E) Predicted binding sites of miR-142 on 3’-UTRs of KRAS, AKT1, PIK3CA, and SHC4 mRNAs from the TargetScan database. Data are shown as mean ± SD of biological repeats (^*^P < 0.05, ^**^P < 0.01, ^***^P < 0.001). SD: standard deviation

To investigate whether miR-142 overexpression influences the expression of EGFR signaling pathway oncogenes in U-87 glioblastoma cells, real-time PCR and western blotting were performed on 5 of its predicted target genes, *KRAS*, *AKT1*, *PIK3CA*, *MAPK8*, and *SHC4* at 72 h after transduction. A dual-luciferase reporter assay of *AKT1* was also performed to see if it is a direct target of miR-142. Real-time PCR showed the reduced expression of all 5 target genes relative to *β2m* internal control by miR-142 transduction compared to the control group. Fold changes were as follows: *KRAS*, 0.0015 ± 0.0008 (^***^P < 0.001), *AKT1*, 0.2758 ± 0.0365 (^**^P < 0.01), *PIK3CA*, 0.0001 ± 0.0001 (^***^P < 0.001), *MAPK8*, 0.0041 ± 0.0008 (^***^P < 0.001), and *SHC4*, 0.0091 ± 0.0014 (^***^P < 0.001) (Figure 4A). Besides, according to the results of western blotting analysis, the protein expression level of these target genes was significantly lower relative to the β-actin internal control by miR-142 transduction in comparison with the scrambled control group. Fold changes were as follows: KRAS, 0.609 ± 0.131 (^**^P<0.01), AKT1, 0.332 ± 0.109 (^***^P < 0.001), PIK3CA, 0.290 ± 0.050 (^***^P < 0.001), MAPK8, 0.721 ± 0.080 (^**^P < 0.01), and SHC4, 0.769 ± 0.077 (^**^P < 0.01) (Figures 4B-4C). Altogether, miR-142 modulated the expression of *KRAS*, *AKT1*, *PIK3CA*, *MAPK8*, and *SHC4*, acting as oncogenes of the EGFR signaling pathway. Besides, the dual-luciferase reporter assay results confirmed the role of AKT1 as a direct target of miR-142. The luciferase activity significantly became less in miR-142 transfected cells by 0.38 ± 0.13 (^**^P < 0.01) in comparison with the control (Figure 4D). According to Figure 4E, *in-silico* prediction by TargetScan tool showed that miR-142 directly controls other oncogenes of the EGFR signaling pathway as well, so it suppresses oncogenes in glioblastoma cells.

## Discussion

The miRNAs are natural oligonucleotides modulating gene expression at the post-transcriptional level. They control physiological processes, such as growth, proliferation, differentiation, and apoptosis by controlling their relevant genes. So, miRNAs have great potential to change the expression of oncogenes in cancer cells and suppress tumor features. Tumor suppressor miRNAs have been used to inhibit proliferation and migration of glioblastoma cells (18-22), and some of them are in clinical trial phases (23).

Several previous studies have addressed the tumor suppressor function of miR-142 in glioblastoma (24-26) or other cancers, such as colorectal (27) and non-small-cell lung carcinoma (28). Qin et al., showed that miR-142 is downregulated in tissues of patients with GBM and its ectopic overexpression in glioblastoma cell lines suppressed cell migration and invasion by targeting Ras-related C3 botulinum toxin substrate 1 (*RAC1*), leading to suppression of matrix metalloproteinases (*MMPs*) (24). In a similar study, Li et al., revealed miR-142 downregulation in patients with GBM. They showed that miR-142 overexpression suppresses cell proliferation and induces apoptosis by targeting high mobility group box protein 1 (*HMGB1*) via the Wnt/β-catenin signaling pathway in glioblastoma cells (25). Consistent with the mentioned studies, our findings approved tumor suppressor role of miR-142 in glioblastoma cells via suppression of cell proliferation and migration and induction of apoptosis and cell cycle arrest and also sheds light on its molecular mechanism of action via regulation of EGFR pathway.

First, miRNA regulators of the EGFR signaling pathway were searched, and it was predicted that miR-142 regulates several oncogenes involved in the EGFR pathway. Most of these oncogenes were overexpressed or mutant in glioblastoma, besides miR-142 was less expressed in glioblastoma tissues vs. normal tissues (Figure 1). EGFR signaling pathway is the most common oncogenic pathway in patients with GBM that causes cell growth (7). Our results showed that predicted target genes of miR-142 from the EGFR pathway, *SHC4*, *KRAS*, *AKT1*, *PIK3CA*, and *MAPK8* become less expressed at mRNA and protein levels by its overexpression via lentiviral transduction. At the same time, proliferation and migration of U-87 glioblastoma cells were reduced, and they were more susceptible to apoptosis and cell-cycle arrest in comparison with the control group.

Also, our findings confirmed that *AKT1* is a direct target of miR-142 via dual-luciferase reporter assay. When EGFR triggers PIK3CA, it activates AKT1 via phosphorylation. There are various functional proteins at downstream of AKT1. For example, AKT1 activates the oncogenic mammalian target of rapamycin (mTOR) that promotes cell growth and proliferation (29). AKT1 also inhibits apoptosis and promotes survival via functioning on Bad and Bim apoptotic proteins. On the other hand, it was observed that miR-142 downregulated SHC4/KRAS/MAPK8 axis either directly or indirectly. MAPK is another oncogenic module downstream of EGFR that induces cell mobility and migration in glioblastoma cells (30). So, it seemed that miR-142 is a tumor suppressor controlling all these oncogenes.

**Table 1:** Predicted target genes of miR-142 in TargetScan and miRWalk databases in common with the EGFR signaling pathway

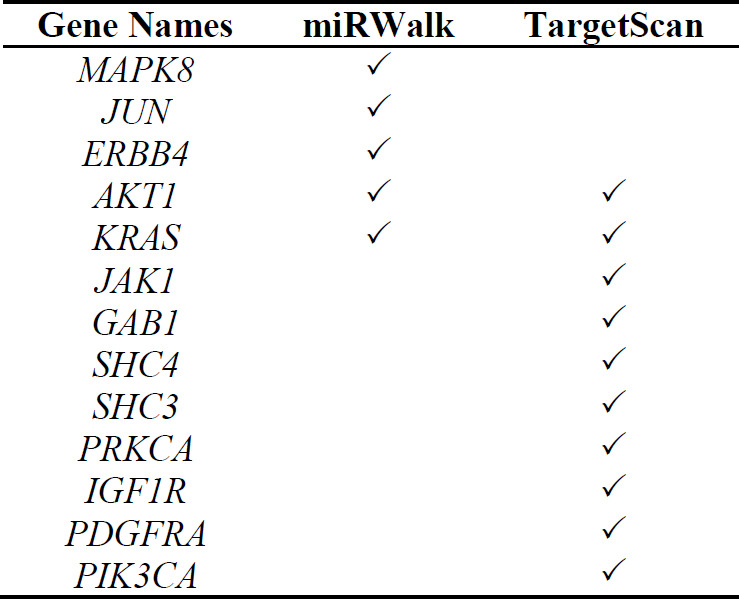

**Table 2:** *Sequences of primers for real-Time PCR of *miR-142* relative to *SNORD47* internal control*

*miR-142* RT Primer	GTCGTATGCAGTGCAGGGTCCGAGGTATTCGCACTGCATACGACAGTAGT
*miR-142* Forward Primer	GGTGGGTCATAAAGTAGAAAG
*SNORD47* RT Primer	GTCGTATGCAGAGCAGGGTCCGAGGTATTCGCACTGCATACGACAACCTC
*SNORD47* Forward Primer	ATCACTGTAAAACCGTTCCA
universal Reverse Primer	GAGCAGGGTCCGAGGT

**Table 3:** *Sequences of primers for real-Time PCR of target genes relative to *β2M* internal control*

**Gene Names**	**Forward Primer**	**Reverse Primer**
*β2M*	ATGCCTGCCGTGTGAAC	ATCTTCAAACCTCCATGATG
*KRAS*	CACAGCAGGTCAAGAGGAG	TTATGGCAAATACACAAAGAAAGC
*AKT1*	TGGCACCTTCATTGGCTAC	GTCTGGATGGCGGTTGTC
*PIK3CA*	CTCCTCTAAACCCTGCTCATC	CATATCTTGCCGTAAATCATCC
*MAPK8*	TTCTGCTGGAATTATTCATCGG	GTCACTACATAAGGCGTCATC
*SHC4*	ACGGAACAAATGGCTTACTG	TTGGATGGACATTACCTATTGC

## Conclusion

In conclusion, our findings revealed that miR-142 has therapeutic potential via suppressing glioblastoma cell growth and invasion and can be used as a regulator of oncogenic EGFR signaling pathway as well.

## Data availability

The datasets used and/or analyzed during the current study are available from the corresponding author on reasonable request.

## Declaration

The authors declare that there is no conflict of interest. 

## Author contribution

The study was conceptualized by EA and MS. The methodology was given by EA and FJA. Formal analysis and investigation were done by FJA and FFA. Writing and original draft preparation was done by FG and EA. Funding acquisition was provided by MS and EA. FG performed experiments.
